# Increased prevalence of erythema multiforme in patients with COVID-19 infection or vaccination

**DOI:** 10.1038/s41598-024-52099-z

**Published:** 2024-02-02

**Authors:** Wafaa Saleh, Hamad Alharbi, Seunghee Cha

**Affiliations:** 1 Oral Medicine, Periodontology, Diagnosis and Oral Radiology Department, Faculty of Dentistry, Mansoura University, Mansoura, 33516 Egypt; 2 Department of Oral and Maxillofacial Surgery, King Abdulaziz University, Jeddah, Saudi Arabia; 3 Oral and Maxillofacial Diagnostic Sciences, University of Florida College of Dentistry, Gainesville, FL 32610 USA; 4 Center for Orphaned Autoimmune Disorders (COAD), University of Florida College of Dentistry, Gainesville, FL 32610 USA

**Keywords:** Immunology, Microbiology, Health care, Health occupations, Medical research

## Abstract

Several reports stated that erythema multiforme (EM) was associated with COVID-19 with detrimental outcomes in patients. However, since most of these are case reports, it is challenging to quantitively assess their associations. Therefore, our study aims to determine the prevalence of EM in the context of COVID-19. The study was designed as a retrospective cross-sectional hospital-based study of registered patients at the University of Florida Health Hospital. The ICD-10 codes for EM, COVID-19 infection, and COVID-19 vaccines were searched in the database. The odds ratio was calculated to assess the risk of EM after COVID-19 infection or vaccination. Our study included 43,547 patients with a history of COVID-19 infection, of whom 92 developed EM. Patients with COVID-19 infection were 6.68 times more likely to have EM than those without COVID-19 (P < 0.0001). Similarly, the risk of developing EM after COVID-19 vaccination was 2.7, significantly higher than the general population (P < 0.0001). The prevalence of EM following COVID-19 infection or vaccination significantly differs from the general population, highlighting the importance of monitoring patients for EM after COVID-19 infection and/or vaccination. It is imperative to disseminate awareness to clinicians and patients regarding the impact of COVID-19 on EM.

## Introduction

Erythema multiforme (EM) is an acute immune mediated mucocutaneous disease affecting the skin and mucous membranes. It presents a wide range of clinical manifestations varying from mild clinical features of the minor form of EM, which may only affect the skin and/or oral mucosa, to severe clinical features of the major form (Steven-Johnson syndrome) or toxic epidermal necrolysis that may involve other organs including the eyes and genital organs. EM is mostly detected in young adult patients as different forms of skin eruptions with or without oral ulcerations^1^.

Various etiological factors of EM were reported, with infection being the major cause. In 90% of patients, EM was detected following viral and fungal infections, while the most common were Herpes simplex virus (HSV) (> 80% of cases), followed by Epstein–Barr virus (EBV) and Mycoplasma pneumoniae. Furthermore, Drugs were reported as a triggering factor for EM including antibiotics, non-steroidal anti-inflammatory drugs, and anticonvulsants. EM may be detected as an adverse drug reaction after vaccination, which was reported following COVID-19 vaccines^2,3^.

The prognosis of EM differs between patients due to the different clinical forms of EM, ranging from 2–3 weeks for the minor form to 4–6 weeks for the major form mentioned earlier. Treatment of EM includes topical and systemic steroids, antihistamines, and analgesics. If the condition is caused by an underlying infection or medication, those underlying causes will need to be identified and treated as well^3–5^.

COVID-19 is a global pandemic that emerged in late 2019. The respiratory illness caused by the novel coronavirus (SARS-CoV-2) has been associated with various other symptoms, including oral and dermatologic diseases^6–9^. Although EM can be triggered by numerous factors, including viral infections, its association with COVID-19 has become a topic of interest in recent years^10^. The effect of COVID-19 on the health of the oral cavity can be primarily affected by the patient's immune system and medication prescribed, as well as the pathogenesis of the virus^11,12^. The oral cavity was reported as a perfect environment for the invasion of the SARS-CoV-2 virus^13^. This virus has an affinity to the angiotensin-converting enzyme 2 receptors located on the oral mucous membranes, salivary glands, and the respiratory tract. To our knowledge, this the first large hospital population-based study, investigating the prevalence and odds ratio of EM after COVID-19 infection or vaccination.

## Materials and methods

### Acquisition of the data

A retrospective cross-sectional study was performed with a focus on the patients registered at the University of Florida Health and Shands Hospital from January 2020 to December 2022. Using the Integrated Data Repository (IDR) i2b2 platform, we extracted the data on COVID-19 (ICD 10-U07.1), EM (ICD 10-L51), and EM after COVID-19 vaccination according to the tenth edition of the International Classification of Diseases (ICD-10). The diagnosis of COVID-19 was confirmed by PCR, while the diagnosis of EM was mainly based on clinical and histopathological features.

We measured the onset of EM within 6 weeks after the diagnosis of COVID-19 infection or receiving COVID-19 vaccination. Cases with a history of exposure to certain triggers of EM were excluded from the current study including HSV (types 1 and 2), EBV, cytomegalovirus, mycoplasma pneumoniae and other triggering infections. Vaccines reported to trigger EM were excluded from the current study including the vaccines of herpes zoster, influenza hepatitis B vaccine, and Measles, Mumps, Rubella Vaccine. The study was limited to the de-identified data, thus not requiring IRB approval for human research.

### Statistical analysis

The patients were divided into six groups; total hospital population, patients with COVID-19 infection, patients with EM disease, patients who received any of the following types of COVID-19 vaccine (AstraZeneca, Janssen (J&J), Novavax, Moderna, and Pfizer), patients with COVID-19 infection and developed EM after the COVID-19 infection, and vaccination. Rates between groups were compared by the prevalence ratios and the odds ratio (OR) was measured. The 95% confidence interval (CI) and P-value for each OR will be tabulated. p < 0.05 is significant.

### Ethics approval and consent to participate

All methods were carried out in accordance with relevant guidelines and regulations (Declaration of Helsinki). The ethical approval and informed consent were waived by Institutional Review Board of university of Florida (UF). Research conducted only using that de-identified data set has been determined by the UF-IRB to not meet the definition of human research, and thus does not require IRB approval.

## Results

We identified 43,547 patients with confirmed COVID-19 infection, while 188,233 received COVID-19 vaccines. 92 cases had EM after COVID-19 infection, compared to 158 patients who developed EM after COVID-19 vaccination. The prevalence of EM occurrence in the COVID-19 group was 0.2%, whereas the prevalence of EM in the general population was 0.03% (CI 5.407–8.699) with P < 0.0001. People with COVID-19 infection were 6.68 times more likely to have EM than those without COVID-19. We found statistically significant differences in the prevalence of EM after COVID-19 across age groups and races (P < 0.0001) (Tables 1 and 2).

**Table 1:** Demographic information on patients with COVID 19, COVID-19 vaccines, EM, and hospital population.

	Total hospital population		COVID-19 patients		COVID-19 vaccinated patients		All EM patients		COVID-19 infection and EM		COVID-19 vaccination and EM	
Number	848,992		43,547		188,233		262		92		158	
Gender (%)												
Males	38,6666	46%	18,718	43%	77,844	41%	123	47%	41	45%	70	44%
Females	46,2326	54%	24,829	57%	110,389	59%	139	53%	51	55%	88	56%
Age (%)												
0–9 years old	68,271	8%	4,578	11%	52	0%	67	26%	28	30%	1	1%
10–17 years old	55,164	6%	3,207	7%	10,246	5%	16	6%	16	17%	20	13%
18–34 years old	165,431	19%	16,500	38%	49,815	26%	40	15%	14	15%	34	22%
35–44 years old	83,057	10%	4,840	11%	19,172	10%	26	10%	6	7%	15	9%
45–54 years old	91,957	11%	4,194	10%	20,233	11%	25	10%	7	8%	25	16%
55–64 years old	135,115	16%	4,315	10%	27,735	15%	31	12%	10	11%	15	9%
65–74 years old	132,210	16%	3,162	7%	33,513	18%	22	8%	3	3%	29	18%
75–84 years old	89,043	10%	2,032	5%	21,596	11%	22	8%	5	5%	19	12%
≥ 85 years old	28,744	3%	719	2%	5868	3%	4	2%	3	3%	1	1%
Race (%)												
American Indian	1030	0.12%	86	0.20%	360	0.19%	3	1%	2	2%	3	2%
Black American	92,714	11%	10,044	23.06%	23,684	12.58%	55	21%	18	20%	32	20%
White	417,813	49%	25,069	57.57%	12,6051	66.97%	166	63%	55	60%	97	61%
Multiracial	6627	1%	723	1.66%	1819	0.97%	3	1%	4	4%	3	2%
Other	314,373	37%	6,100	14.01%	27,536	14.63%	35	13%	13	14%	23	15%

**Table 2:** Odds ratio for occurrence of EM with COVID-19 infection.

Comparisons	OR	95% Wald confidence limits		P-value
EM vs. non-EM	6.68	5.407	8.699	P < 0.0001
Males vs. females	0.96	0.637	1.450	0.42
Age group 18–34 vs. 0–17	4.2	2.308	7.686	P < 0.0001
Age > 34 vs. age < 34	3.3	2.166	5.051	P < 0.0001
Black Americans vs. other races	4.69	2.3	9.582	P < 0.0001
White vs. other races	3.18	1.739	5.826	P < 0.0001

Our study detected different types of EM, including 49 patients with EM minor, 115 with Steven Johnson syndrome, 56 with toxic epidermal necrolysis, and 42 with a history of Stevens Johnson syndrome/toxic epidermal necrolysis overlap syndrome.

People who received a COVID-19 vaccination were 2.7 times more likely to develop EM. The prevalence of EM after COVID-19 vaccination was 0.08% compared to 0.03% in the general population (CI 2.234–3.317) with P < 0.0001. We found statistically significant differences in the prevalence of EM after COVID-19 vaccination across races (P < 0.0001) (Tables 1 and 3).

**Table 3:** Odds ratio for occurrence of EM with COVID-19 vaccination.

Comparisons	OR	95% Wald confidence limits		P-value
EM vs. non-EM	2.7	2.234	3.317	P < 0.0001
Males vs. females	0.95	0.695	1.302	0.37
Age group 18–34 vs. 0–17	1.2	0.701	2.081	0.24
Age > 34 vs. age < 34	0.97	0.703	1.35	0.44
Black Americans vs. other Races	4.7	2.761	8.0	P < 0.0001
White vs. other races	3.173	2.014	5	P < 0.0001

## Discussion

In the present study, the prevalence of EM was significantly increased in the COVID-19 group. COVID-19 patients were 6.68 times to have EM than people who didn’t experience COVID-19. It is important to note that this study does not establish a causal relationship between COVID-19 and EM due to its cross-sectional design^10^. However, it is possible that COVID-19 may act as a trigger for EM, as previous studies have reported an association between the two^14^. For example, one study found that 28% of COVID-19 patients with acute palmoplantar skin lesions had EM-like lesions.

The majority of EM cases after COVID-19 in our study were detected in patients younger than 34 years old (63%) while 80% of them were children with age range between 1 and 17 years old. These findings align with a previous study, mentioned earlier, reporting that 28% of patients with COVID-19-related palmoplantar skin lesions having EM-like lesions fell into the age ranges between being 1 and 29 years old^14^. Another report by Bapst et al*.*^15^ reported the case of a 13-year-old child who developed EM as the initial sign of multisystem inflammatory syndrome after COVID-19 infection. Palaia et al*.*^16^ also described EM as an early symptom of COVID-19 in a 30-year-old woman without any other multi-organ symptoms despite being immunocompetent.

Our hospital established a comprehensive approach to diagnose EM through clinical evaluation of the characteristic features of EM including the target lesion with concentric rings and skin rashes. However, if it was difficult to clinically diagnose the case, skin biopsy was obtained to confirm the diagnosis based on histological features, including the presence of keratinocyte apoptosis. However, we acknowledge the potential challenges in differentiating EM from conditions like HSV oral lesions and mycoplasma-induced mucositis due to the similarity in presentation. To address this, a rigorous diagnostic process was used including the clinical assessment, histopathological examination and laboratory testing including PCR for HSV, mycoplasma, and others to confirm or exclude these specific etiologies.

The pathophysiological mechanism of EM after COVID-19 infection may be due to targeting the SARS-CoV-2 antigens in the skin by the lymphocytes inducing a hypersensitivity reaction with apoptosis of the epithelial cells similar to what was reported for EM associated with other infections^17^. Besides the viral cytopathic effect, it was reported that SARS-CoV-2can induce Type II and Type IV hypersensitivity reactions. The cytokine storm which refers to the release of cytokines from host cells was linked to COVID-19 infection. All these factors may explain the possible relationship between EM and COVID-19^18,19^.

The occurrence of EM following COVID-19 vaccination may indicate an adverse drug reaction. However, the causal relationship between the vaccine and EM has not yet been established. Nevertheless, it is not uncommon for medications to trigger this disease^1,17^. We observed a prevalence of EM after vaccination of 0.08% in our study, which was significantly higher than the general population (P < 0.0001). Although age does not seem to affect the occurrence of EM after vaccination, we found significant differences between different races. Interestingly, females reported a higher percentage of EM after COVID-19 infection as well as COVID-19 vaccination.

There have been reports of several cases of EM following COVID-19 vaccinations. A study reported three cases of EM occurring within two to four days after the first dose of the Pfizer-BioNTech COVID-19 vaccine^20^. The authors of the study suggested a possible association between the vaccine and the development of EM. Similarly, in another report, four patients developed oral and dermatologic lesions of EM after receiving the Pfizer- BNT162b2 vaccine, despite having no history of erythema-like or herpetic manifestations^21^. In addition, Kong et al. detected EM-like lesion after the second dose of mRNA-1273 (Moderna) COVID-19 vaccine^22^. Although there have been reports of EM after COVID-19 vaccination, the incidence appears to be very low, and the exact cause of this phenomenon is still being investigated^20^. Interestingly, Lavery et al*.* reported a recurrence of EM after BNT162b2 vaccination in a female patient who had already been affected by the disease in 2018^23^. The patient had bilateral cutaneous erythematous plaques on the hands and feet without oral involvement.

COVID vaccination offers crucial relative risks and benefits. It significantly reduces the risk of severe COVID-19 illness, hospitalization, and death while presenting minimal risks compared to the disease itself. Vaccination remains a pivotal tool in curbing the pandemic's impact and safeguarding public health. We noticed that our study found a higher incidence of vaccine-induced EM compared to the available literature on case reports and small cohorts. However, we believe this is an important aspect of our findings, as it highlights a potential discrepancy between real-world data and previously reported cases. This raises the need for further investigation into the underlying mechanisms and risk factors, which can contribute to a more comprehensive understanding of vaccine side effects^24,25^.

We acknowledge that our patient cohort primarily consists of individuals who developed COVID-19 while hospitalized. This selection may not fully represent the broader community population where COVID-19 prevalence might differ. Our aim was to investigate and analyze the outcomes of COVID-19 cases within a hospital setting.

While the COVID-19 Vaccination campaign is progressing, the knowledge about the probable side effects is growing and their adverse events are new scopes in medicine. The exact mechanism by which the COVID-19 vaccine triggers EM is not yet fully understood. However, it is believed to be related to the immune response generated by the vaccine, which can result in the development of an immune-mediated skin reaction. Similar to other vaccines, the expression of an antigen on keratinocyte may cause T-cell activation. Vaccination stimulates the immune system and causes T cell polarization^26,27^. Furthermore, Sahin et al*.* reported that the BNT162b2 vaccine induced a coordinated humoral and cellular adaptive immunity^28^. Others suggest that the vaccine may trigger the reactivation of a previous infection or an underlying autoimmune disorder^21,28^.

The limitation of the study includes that the i2b2 IDR provides the total number of patients who meet the selected search criteria. In other words, the search outcome consists of the total number of patients with a certain disease rather than any individual patient data. In addition, certain drugs received by COVID-19 positive patients may trigger EM however, the i2b2 IDR provides the total number of patients who meet the selected criteria (the total number of positivity or negative cases for particular clinical/ laboratory parameters searched) rather than providing the medication received by each patient. Therefore, there is no way to know the drugs received by each patient during COVID-19 infection before the appearance of EM. Another limitation of our study is that the cross-sectional design can’t provide the true causality of the diseases. We acknowledge that there might be potential biases in data collection and reporting. So, further research is required to confirm our findings and explore the potential mechanisms behind the observed differences in EM incidence after COVID-19 vaccination.

In conclusion, EM is a skin condition that can be triggered by various factors such as infections, medications, and vaccines. There have been reports of EM occurring in patients after COVID-19 infection or vaccination, indicating that the virus may serve as a trigger for this condition or host immune reactions to the vaccination may lead to EM. respectively. Further research is necessary to establish the association between COVID-19 and EM and develop more effective treatment protocols. It is crucial for clinicians to be aware of the link between the two conditions, which can facilitate the diagnosis and management of both conditions. Early recognition of oral and dermatologic diseases linked to SARSCoV-2 infection by informed clinicians will allow the early detection of COVID-19 infection and/or timely intervention to prevent a serious sequela of both conditions.

## Data Availability

All data generated or analyzed during this study are included in this published article.

## References

[1] Grünwald P, Mockenhaupt M, Panzer R, Emmert S (2020). Erythema multiforme, Stevens-Johnson syndrome/toxic epidermal necrolysis—Diagnosis and treatment. J. Dtsch. Dermatol. Ges..

[2] Rizo-Potau D, Marti-Marti I, Fustà-Novell X (2021). Erythema multiforme. Med. Clin..

[3] Samim F, Auluck A, Zed C, Williams PM (2013). Erythema multiforme: A review of epidemiology, pathogenesis, clinical features, and treatment. Dent. Clin. North Am..

[4] Sola CA, Beute TC (2014). Erythema multiforme. J. Spec. Oper. Med. Fall.

[5] Trayes KP, Love G, Studdiford JS (2019). Erythema multiforme: Recognition and management. Am. Fam. Phys..

[6] Erbaş GS (2022). COVID-19-related oral mucosa lesions among confirmed SARS-CoV-2 patients: A systematic review. Int. J. Dermatol..

[7] Martín Carreras-Presas C, Amaro Sánchez J, López-Sánchez AF, Jané-Salas E, Somacarrera Pérez ML (2021). Oral vesiculobullous lesions associated with SARS-CoV-2 infection. Oral Dis..

[8] Daneshgaran G, Dubin DP, Gould DJ (2020). Cutaneous manifestations of COVID-19: An evidence-based review. Am. J. Clin. Dermatol..

[9] Saleh W, Ata F, Elashry MM (2021). Is COVID-19 infection triggering oral herpes zoster? A case report. SAGE Open Med. Case Rep..

[10] Bennardo L (2021). Erythema multiforme and COVID-19: What do we know?. Medicina.

[11] Fidan V, Koyuncu H, Akin O (2021). Oral lesions in Covid 19 positive patients. Am. J. Otolaryngol..

[12] Saleh W, Eman SH, Halim GA, Ata F (2021). Oral lichen planus after COVID-19, a case report. Ann. Med. Surg..

[13] Xu H (2020). High expression of ACE2 receptor of 2019-nCoV on the epithelial cells of oral mucosa. Int. J. Oral Sci..

[14] Fernandez-Nieto D (2020). Characterization of acute acral skin lesions in nonhospitalized patients: A case series of 132 patients during the COVID-19 outbreak. J. Am. Acad. Dermatol..

[15] Bapst T, Romano F, Müller M, Rohr M (2020). Special dermatological presentation of paediatric multisystem inflammatory syndrome related to COVID-19: Erythema multiforme. BMJ Case Rep..

[16] Palaia G (2022). Erythema multiforme as early manifestation of COVID-19: A case report. Pathogens..

[17] Sokumbi O, Wetter DA (2012). Clinical features, diagnosis, and treatment of erythema multiforme: A review for the practicing dermatologist. Int. J. Dermatol..

[18] Icenogle T (2020). COVID-19: Infection or autoimmunity. Front. Immunol..

[19] Dotan A (2021). The SARS-CoV-2 as an instrumental trigger of autoimmunity. Autoimmun. Rev..

[20] McMahon DE (2021). Cutaneous reactions reported after Moderna and Pfizer COVID-19 vaccination: A registry-based study of 414 cases. J. Am. Acad. Dermatol..

[21] Petruzzi M, Galleggiante S, Messina S, Della Vella F (2022). Oral erythema multiforme after Pfizer-BioNTech COVID-19 vaccination: A report of four cases. BMC Oral Health..

[22] Kong J (2021). Bullous drug eruption after second dose of mRNA-1273 (Moderna) COVID-19 vaccine: Case report. J. Infect. Public Health..

[23] Lavery MJ, Nawimana S, Parslew R, Stewart L (2021). A flare of pre-existing erythema multiforme following BNT162b2 (Pfizer-BioNTech) COVID-19 vaccine. Clin. Exp. Dermatol..

[24] Verdecia M (2021). COVID-19 vaccine platforms: Delivering on a promise?. Hum. Vaccin. Immunother..

[25] Fathizadeh H (2021). SARS-CoV-2 (Covid-19) vaccines structure, mechanisms and effectiveness: A review. Int. J. Biol. Macromol..

[26] Loche F, Schwarze HP, Thedenat B, Carriere M, Bazex J (2000). Erythema multiforme associated with hepatitis B immunization. Clin. Exp. Dermatol..

[27] Egan KP, Wu S, Wigdahl B, Jennings SR (2013). Immunological control of herpes simplex virus infections. J. Neurovirol..

[28] Sahin U (2021). BNT162b2 vaccine induces neutralizing antibodies and poly-specific T cells in humans. Nature..

